# A101 SCALES EVALUATING THE CLEANLINESS AND MUCOSAL VISIBILITY OF THE UPPER GI TRACT: A SCOPING REVIEW

**DOI:** 10.1093/jcag/gwae059.101

**Published:** 2025-02-10

**Authors:** D Tham, N Gimpaya, W Tran, A Ibrahim, Y Verma, S Grover

**Affiliations:** Scarborough Health Network Research Institute, Toronto, ON, Canada; Scarborough Health Network Research Institute, Toronto, ON, Canada; Scarborough Health Network Research Institute, Toronto, ON, Canada; Scarborough Health Network Research Institute, Toronto, ON, Canada; Scarborough Health Network Research Institute, Toronto, ON, Canada; Scarborough Health Network Research Institute, Toronto, ON, Canada

## Abstract

**Background:**

Esophagogastroduodenoscopy (EGD) requires clear mucosal visualization for detecting lesions in the upper gastrointestinal (UGI) tract. Historically, studies have evaluated UGI cleanliness using non-validated scales. The validation of scoring methods for UGI cleanliness is necessary to be able to document EGD quality in a standardized and reproducible manner. Recently, there have been multiple published studies which describe the development of novel cleanliness scales for EGD, and there is a need to synthesize the currently available data.

**Aims:**

To summarize and compare novel UGI cleanliness scales for EGD.

**Methods:**

A literature search of MEDLINE and Web of Sciences from inception to September 2024 identified randomized and cohort studies that used UGI cleanliness scoring systems. Two reviewers screened abstracts/articles and extracted data. Baseline study characteristics and information about cleanliness scales were extracted. The primary outcome was characteristics and validity evidence for UGI cleanliness scales.

**Results:**

Of 1729 articles screened, 65 were analyzed (Figure 1). 53 studies developed scales for EGD and 11 for capsule endoscopy. 27 articles described novel scales that were not based on prior studies. The primary objectives of these studies were to develop or validate an UGI cleanliness scale (5), evaluate devices (5), or assess intervention efficacies (17). Table 1 summarizes studies that developed a UGI cleanliness scale as a primary aim.

Among the 38 studies which created or utilized UGI cleanliness scales based on other studies, the most highly cited scale (13) was proposed by Kuo et al. in 2002. This 4 point scale (1=optimal visibility, 4=poor visibility) evaluated the gastric antrum, lower body, upper body, and fundus. No validation studies were performed, but scores between multiple endoscopists showed a correlation with each other (r = 0.59-0.78, P < 0.001).

**Conclusions:**

In this review, we have synthesized the current body of literature on UGI scales, highlighting their testing and validity. Future studies should compare the scores from UGI scales with clinical outcomes to determine the utility of standardized scales in improving EGD quality.

Table 1: Summary of studies with UGI cleanliness scale validation/development as the primary outcome.

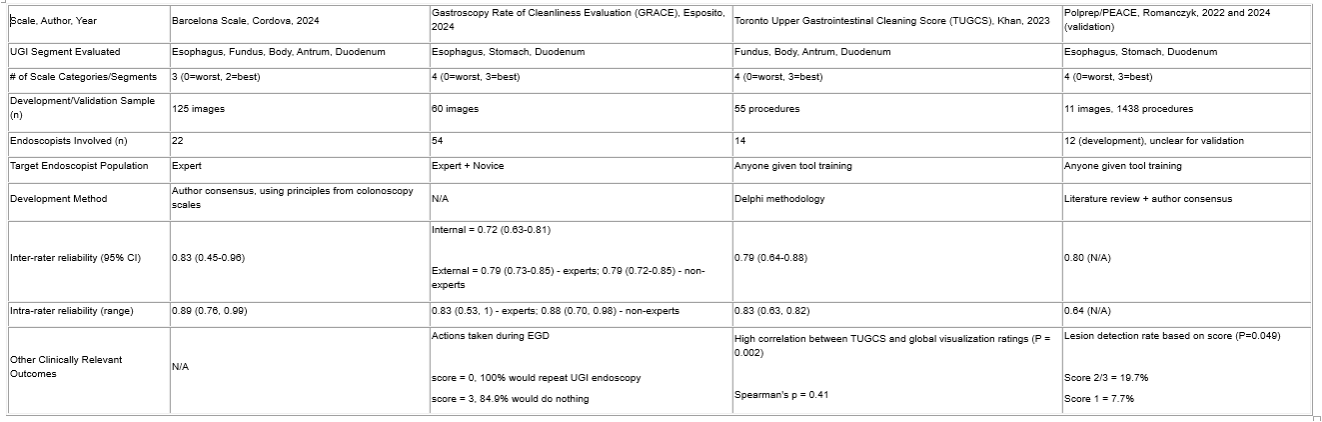

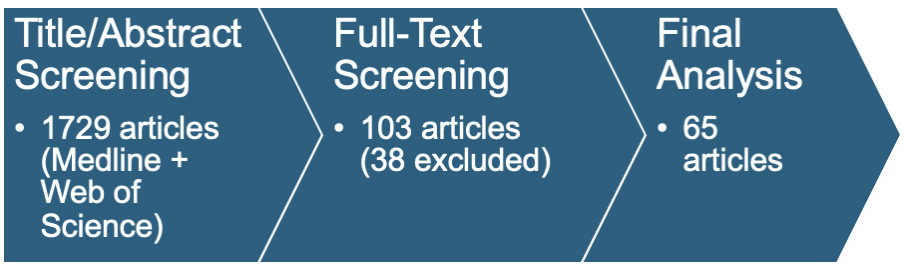

**Funding Agencies:**

None

